# Effect of segmentation techniques (manual versus semiautomatic) on the accuracy of the linear measurements driven from the reconstructed mandibular condyle using CBCT

**DOI:** 10.1186/s12903-026-07947-x

**Published:** 2026-03-17

**Authors:** Enas El Saket, Enas Anter, Ahmad M. Abd El Samad

**Affiliations:** Oral and Maxillofacial Radiology Department, Faculty of Dentistry, Cairo University, Giza, Egypt

**Keywords:** CBCT, Condyle, Manual segmentation, Semiautomatic segmentation

## Abstract

**Background:**

Assessment of the effect of segmentation technique (manual Vs semiautomatic) on the accuracy of linear measurements of the CBCT reconstructed 3D mandibular condyle compared to the real measurements of the actual condyle.

**Materials and methods:**

fifteen dry mandibular with 30 condyles were included in this study, where condylar real linear measurements (L1, L2& L3) were assessed using a digital caliper. Then each mandible was assembled to a skull in a normally closed TMJ position, this assembly was scanned using Planmeca Promax 3D Mid CBCT machine with a standard imaging protocol. Later, *3D Slicer* open-source segmentation software was used for manual and semiautomatic segmentation of mandibular condyles from the CBCT images. Afterward, the same linear measurements were assessed again on the CBCT reconstructed 3D condyles. Statistical analysis showed a normal distribution of the numerical data of all linear measurements, so repeated measures ANOVA followed by Bonferroni post hoc test were used to compare the measurements on the 3D segmented CBCT condyles to their corresponding real measurements. Reliability analysis using the intraclass correlation coefficient (ICC) test was also adopted.

**Results:**

There was no statistically significant difference in linear measurement errors between manually and semiautomatically segmented condyles. The manual and semiautomatic segmented condylar width(L1) measurements showed excellent agreement with real measurements, while length (L2) measurements showed good agreement, and height (L3) measurements had poor agreement.

**Conclusion:**

We're using the Semiautomatic segmentation technique provides a reliable and time-saving segmentation approach that can yield accurate results compared to manual segmentation.

**Trial registration:**

The study protocol was registered on www.clinicaltrials.gov (ID: NCT05435417).

## Background

Over the years, three-dimensional (3D) radiographic techniques have been developed to assist clinicians in the analysis of complex craniofacial structures like the temporomandibular joint (TMJ) [1] The gold standard imaging modalities for the TMJ were magnetic resonance imaging (MRI) and computed tomography (CT). Although CT gives superior evaluation of the TMJ osseous component than MRI, it still provides a high radiation dose. Subsequently, Cone beam computed tomography (CBCT) has been considered the optimum technique to evaluate the osseous component of the TMJ due to its high spatial resolution and low radiation dose [2].

CBCT offers 3D volumetric assessment of the condyle, which is a complex anatomical structure of great importance in maxillofacial dentistry. The mandibular condyle significantly affects the mandibular growth, therefore morphological and dimensional condylar changes can affect craniofacial growth. Furthermore, temporomandibular disorder (TMD), as well as various malocclusions, can be the consequences of improper mandibular condylar growth and development [2, 3]. Morphological and histological changes in the temporomandibular joint can be caused by age progression and functional disorders as well [4].

With the development of digital dentistry and the rise of variable planning software, an easy and detailed 3D visual assessment of the condyle became possible with the segmentation process. This process separates a specific tissue from the background into a reconstructed homogeneous structure [5]. Different segmentation techniques have been used to segment the mandibular condyle, as the manual technique, in which the clinician demarcates the condylar outline in each slice to produce a segmented 3D reconstructed condyle. While in the automatic technique, the operator chooses a specific range of threshold intervals to guide the segmentation. Nonetheless, the automatic technique is fast and convenient, but a major drawback emerges, which is the precise delineation of complex structures [5]. On the other hand, semiautomatic segmentation combines automatic and manual segmentation using the binary threshold-based volume or the region-growing algorithm. The characteristic advantage of the semiautomatic technique over the manual technique is clinically essential time saving. However, manual segmentation was supposed to provide higher accuracy in detecting anatomical structures with low-density or non-apparent borders, especially complex structures such as the TMJ [2, 5].

Regardless of the technique used, the accuracy of the segmentation might also be dependent on other variables like the quality of the scan used, which is mainly a function of the CBCT machine, and the exposure parameters used for scan production, as well as the software used in the segmentation process [2, 5–7], even the object being segmented [1, 8]. Previous studies reported that the condyle is one of the most challenging structures for segmentation in the maxillofacial region, where the accuracy of its segmentation could be affected by the complexity of its shape, its low bone density and how close it is located to the adjacent anatomical structure of the same radio-density [9].

For that, this study was planned to assess the effect of different segmentation techniques, manual and semiautomatic, on the accuracy of linear measurements of the 3D reconstructed mandibular condyle from CBCT scans, as compared to the actual physical linear measurements of the mandibular condyles as a gold standard.

## Methods

This study was conducted after the approval of the Research Ethics Committee of the Faculty of Dentistry, Cairo University. The study protocol was registered on www.clinicaltrials.gov (ID: NCT05435417) The study is an in vitro experimental validation study using dry skulls.

A power analysis was designed to have adequate power to apply a two-sided statistical test of the null hypothesis that semiautomatic segmentation of CBCT images will provide the same accuracy of reconstructed mandibular condyle as the manual segmentation of these images. By adopting an alpha level of (0.05) a beta of (0.2) i.e. power = 80% and an effect size (d) of (0.543) calculated based on the results of a previous study by Kim et al.[1], the predicted sample size (n) was found to be (29) condyles.

### Study participants

A total of 30 condyles were included in this study. Fifteen skulls and mandibles were recruited from the Anatomy Department, Faculty of Medicine, Cairo University. Sample preparation, direct measurements, radiographic examination, and data collection were performed in the Oral and Maxillofacial Radiology Department, Faculty of Dentistry, Cairo University.

Human skulls and mandibles were recruited according to the following eligibility criteria: mandibles with sound condyles and skulls with sound glenoid fossae were included in the study. The Presence of any fracture, pathological lesion, or skeletal deformity in the condylar area was considered a reason for exclusion.

*For the reference test application *certain anatomical landmarks were identified on the dry mandibular condyles to act as a reference for the linear analysis of the condyle according to García-Sanz et al. [10]. The reference points were: *Anterior point*: The most anterior extent of the mandibular condyle, *Posterior point*: The most posterior extent of the mandibular condyle, *Lateral point*: The most lateral extent of the mandibular condyle, *Medial point*: The most medial extent of the mandibular condyle and *Superior point*: The most superior aspect of the mandibular condyle.

**Fig. 1:**
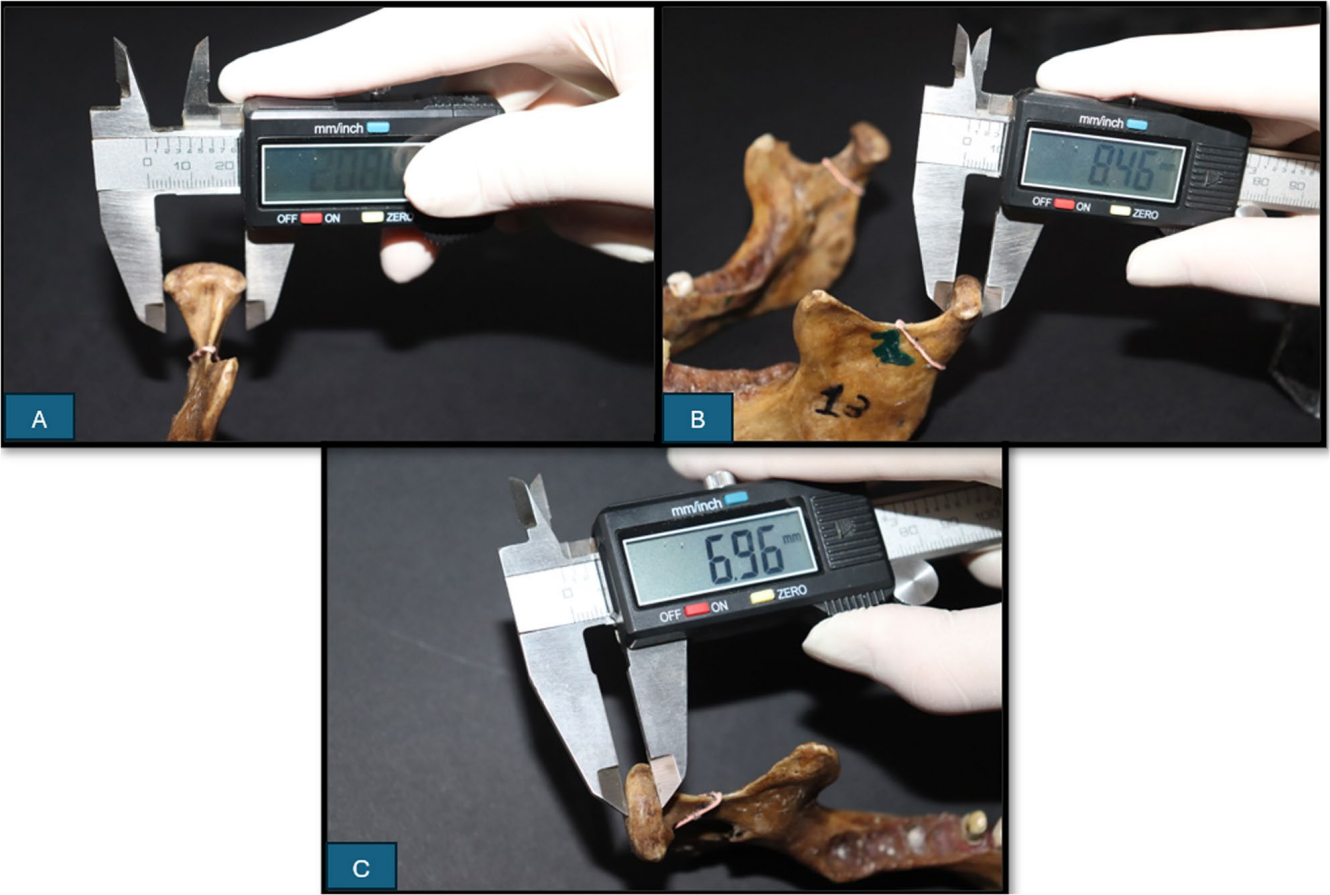
Photographs showing the actual real condylar measurements: **A:** A photograph showing the measurement of condylar width by using a digital caliper as the distance between lateral and medial points (L1). **B:** A photograph showing measurement of condylar length by using a digital caliper as the distance between anterior and posterior points landmarks (L2).**C:** A photograph showing the measurement of condylar height by using a digital caliper as the distance of the perpendicular line traced from the superior point on the condyle to line 1 (L3). A caliper was used to identify the most protruded lateral, medial, anterior, and posterior points

Then three actual linear measurements (gold standard measurements) were taken directly on the dry mandibles using a high precision sliding electronic digital caliper (Radioshack^®^, China) in millimeters, as follows: *Line 1 (L1)*: Condylar width measured as the distance between lateral and medial points (Fig. 1.A). *Line 2 (L2)*: Condylar length measured as the distance between anterior and posterior points (Fig. 1B). *Line 3 (L3)*: Condylar height measured as the distance of the perpendicular line traced from the superior point on the condyle to line 1 (Fig. 1.C). Each real measurement was assessed twice by the principal investigator with two weeks interval, and the average was considered the gold standard.

To reduce variability in landmark identification on irregular condylar surfaces, a caliper was used to identify the most protruded lateral, medial, anterior, and posterior points, with each landmark defined as the first point of contact between the caliper surface and the condyle.

**Fig. 2:**
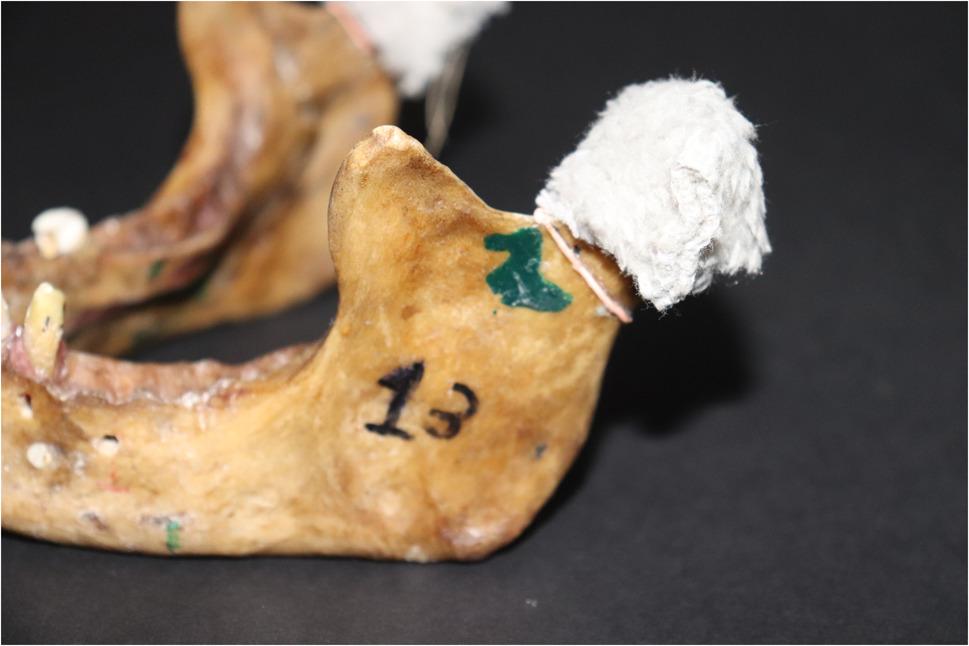
A photograph showing the piece of cloth used to separate between the condyle and the glenoid fossa.

*For the Index Test Application*, each mandibular condyle was fitted with its corresponding glenoid fossa in the skull; this position was secured using wrapping film to hold them together firmly. To simulate the articular disc between the condyle and the fossa, a piece of cloth was placed to separate them, giving the radiographic appearance of the articular disc (radiolucent line separating the two radiopaque surfaces) (Fig. 2).

**Fig. 3:**
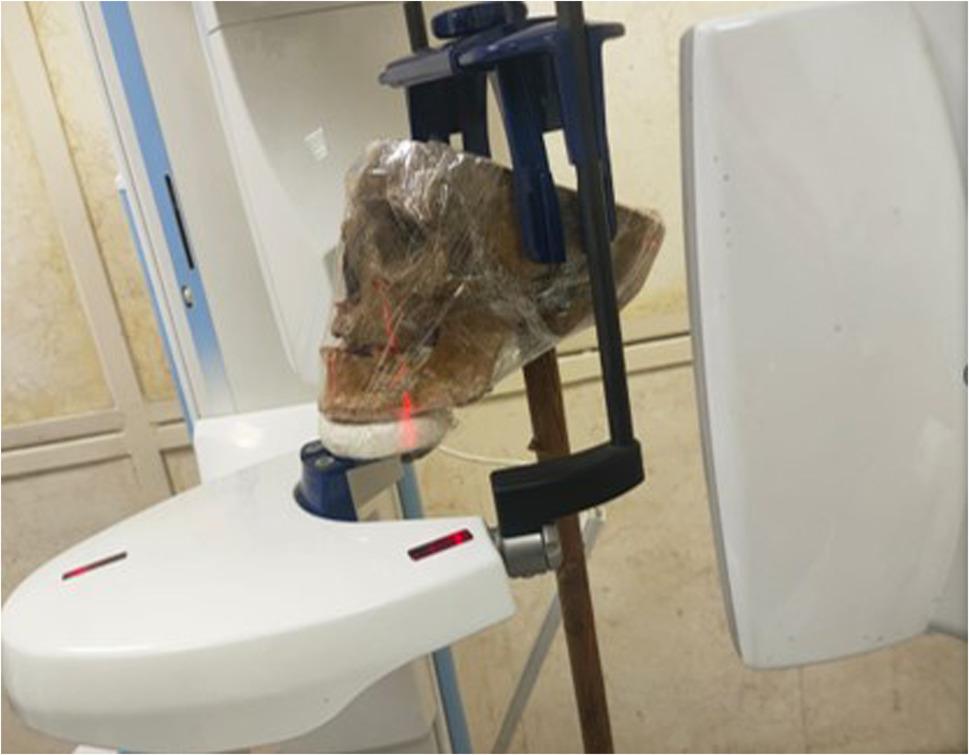
A photograph showing the position of the assembled skull on the CBCT machine

CBCT scanning was done using Planmeca Promax 3D Mid* CBCT machine (Planmeca, Helsinki, Finland). Each skull and mandible assembly were properly positioned in the machine, with the help of the built-in plastic positioning device and extra support gained from a long wooden stick placed with one end just below the external occipital protuberance of the skull and the other one reaching to the floor for stabilization (Fig. 3). The assembly was properly oriented using the laser beam indicators of the machine such that the vertical laser beam coincided with the midsagittal plane (perpendicular to the floor) and the horizontal laser beam coincided with the Frankfort horizontal plane (parallel to the floor). Each skull & mandible assembly was scanned using the standard imaging protocol with Kilovoltage: 90 kV., Milliampere: 8 mA, Field of view: 20 × 6 cm, Voxel size: 0.4 mm, Exposure time: 13.5s.

The generated CBCT images of the assembled skulls and mandibles were imported as “multiple DICOM files” format to 3D slicer version 5.8.1 open-source segmentation software (https://www.slicer.org/). Then, manual segmentation (Index test 1) and semiautomatic segmentation (Index test 2) of the condyles were done using this software.

### Index test 1: manual segmentation of the condyle from CBCT images

On the 3D slicer software, axial slices were scrolled until the crosshair line (red) representing the level of the axial plane in the coronal and sagittal views was set at the most superior point of the condyle. Then the “Segment Editor” module was activated, and the “Add” option was selected to create a new segment (Segment 1), which was then renamed to the condyle number (for example, C1).

**Fig. 4:**
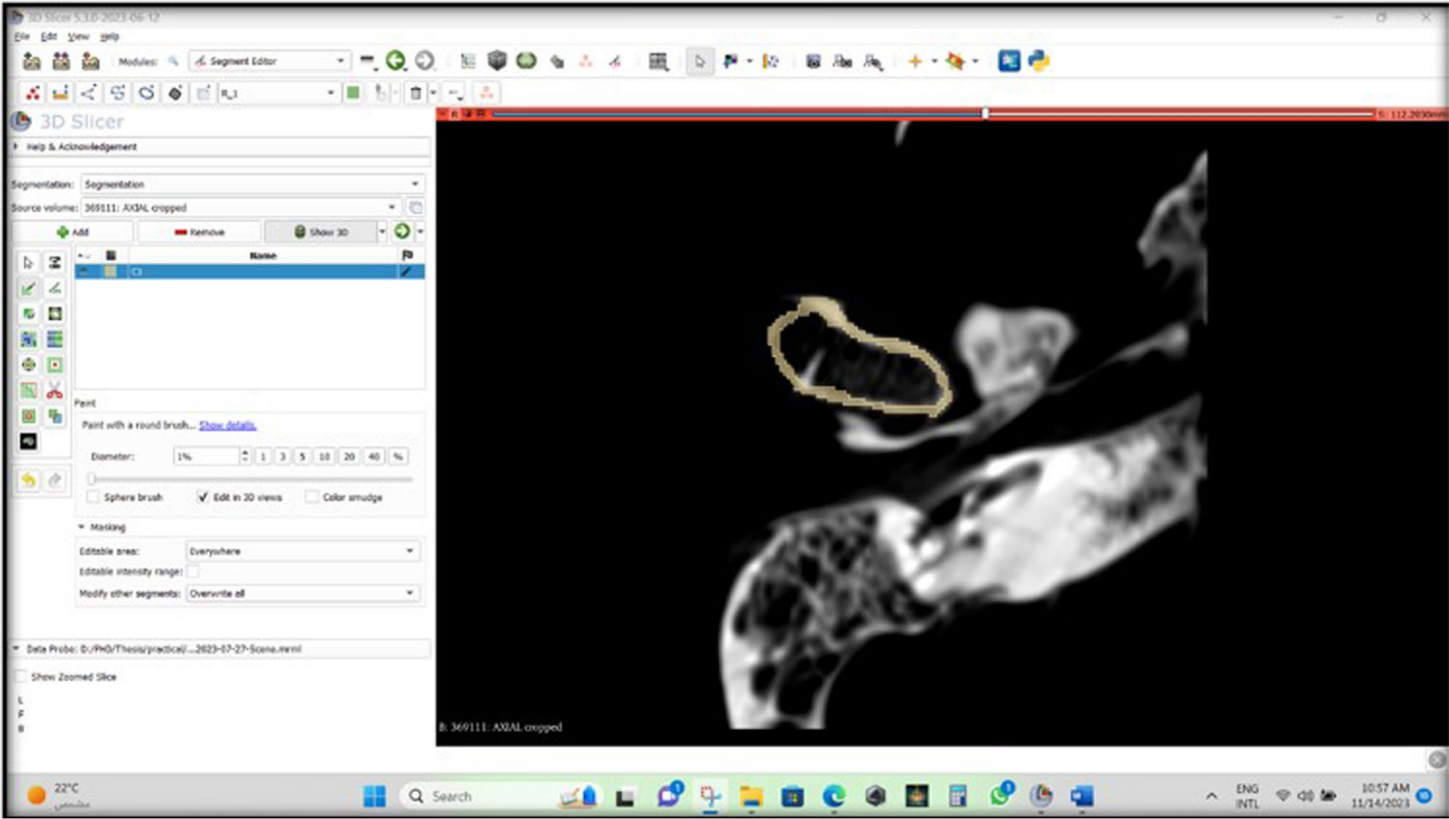
A screenshot from the software showing that the “Paint” option was selected, and the diameter of the brush was set at 0.7 mm to precisely delineate the condylar outline

**Fig. 5:**
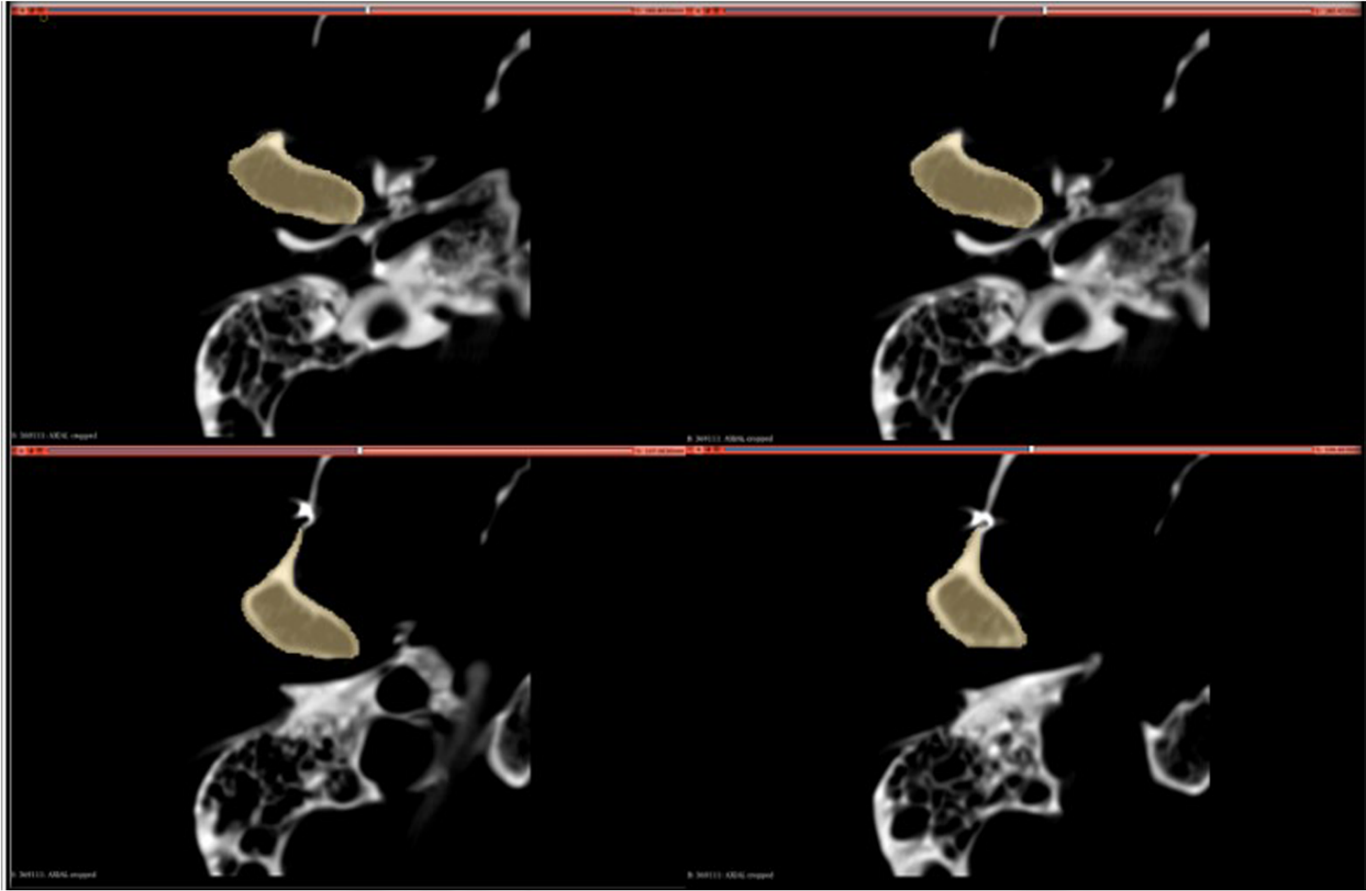
A screenshot from the software showing the application of the paint brush in each slice in the axial plane till the whole condylar volume was painted

Thereafter, condylar outline delineation started using a small brush of 0.7 mm diameter (Fig. 4). Then, a larger brush diameter was used to fill in the whole condyle in each slice in the axial plane until the whole condylar volume was painted (Fig. 5).

**Fig. 6:**
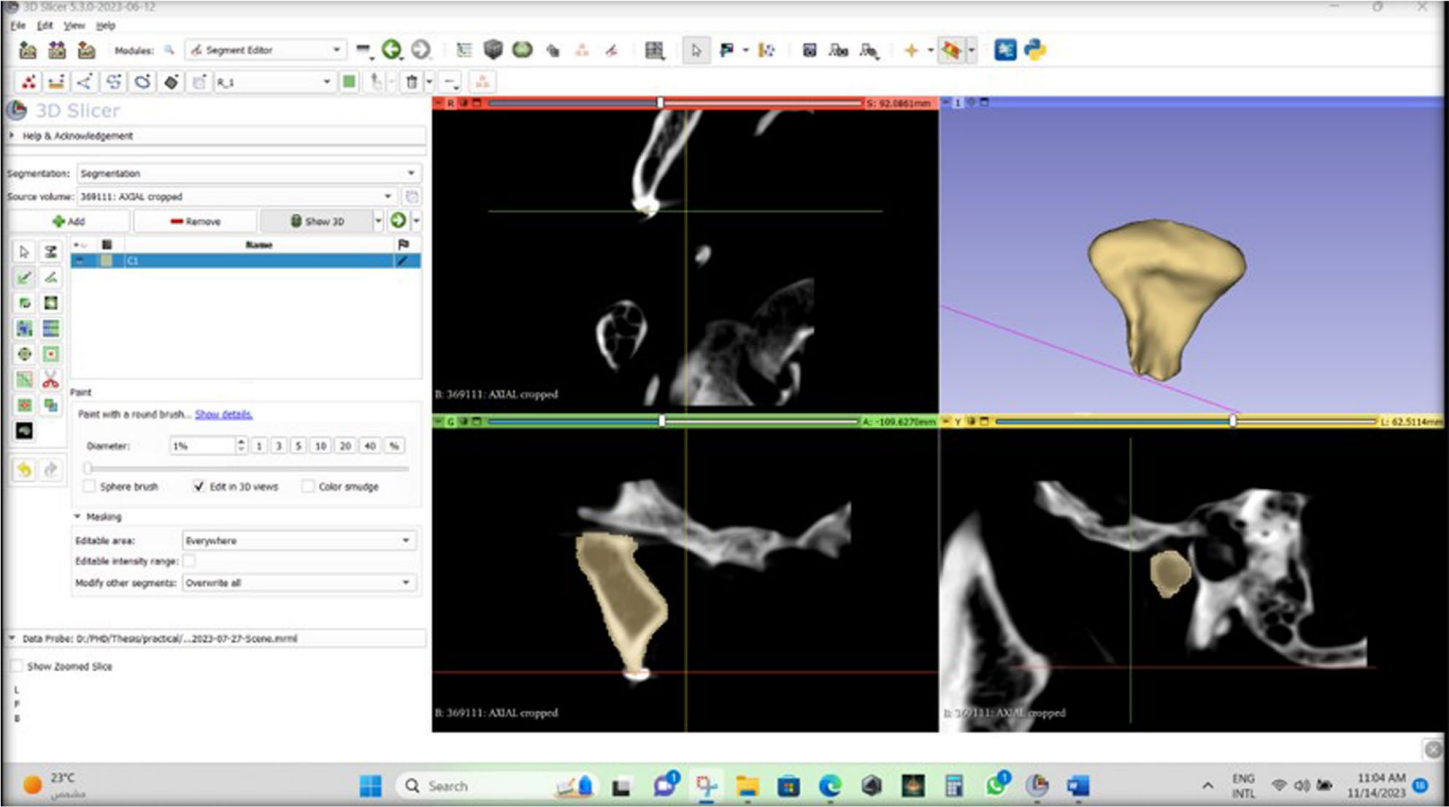
A screenshot from the software showing that the “Show 3D” option was selected to show the segmented condylar volume

Any mishaps could be edited using the “erase” option. Finally, the “Show 3D” option was selected to show the segmented condylar volume **(**Fig. 6**).**

### Index test 2: semiautomatic segmentation of the condyles from CBCT images

The first three steps adopted in the manual segmentation were repeated, then two segments were created, one of which represents the structure targeted for segmentation, while the other segment represents the background signal that should be separated from the targeted structure. The two segments were renamed to condyle number (C1 surrounding) and (C1).

**Fig. 7:**
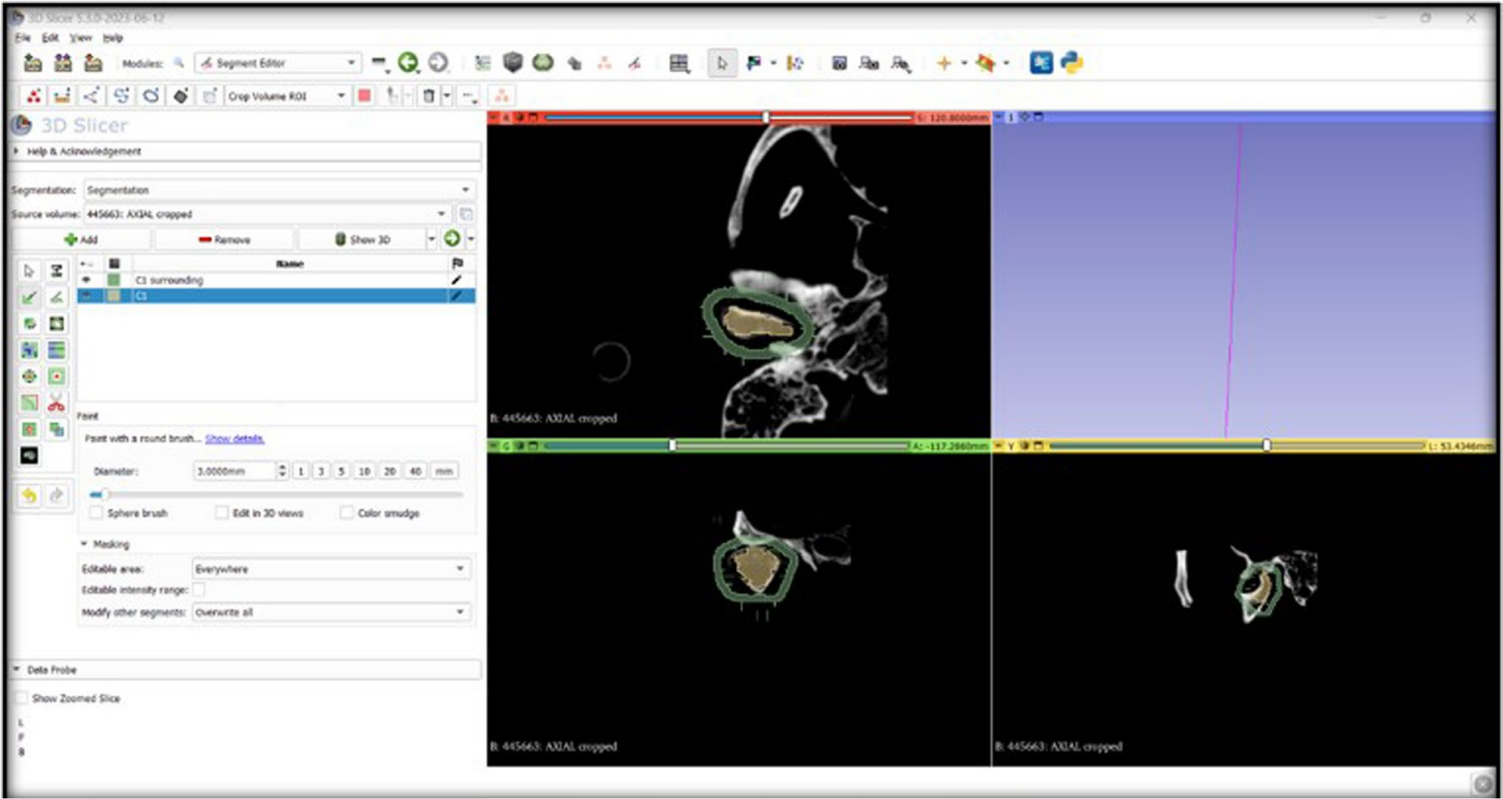
A screenshot from the software showing that the slices of the condyle were painted randomly (C1) till the level of the gutta-percha corresponding to the real condyle, and the surroundings were painted (C1 surrounding). This was done in all planes (axial, coronal, and sagittal). The two segments were painted with two different colors

Afterwards, the slices of the condyle were painted randomly with a 3 mm diameter brush (C1), and the surroundings were also painted (C1 surrounding). This was done in all planes (axial, coronal, and sagittal). The two segments were painted with two different colors **(**Fig. 7**).** Thereafter, the “Grow from seeds” option was selected, followed by the “Initialize” and the “Apply” buttons. A wide range of grey values was used in the “Grow from seeds” method. Finally, the 3D condylar volume was created. Throughout this work, some discrepancies occurred, and the software’s built-in options helped in their modification thanks to the “Paint”, “Erase”, and “Smoothening” options. These options were only used to erase the overextended segmentation volume and not affect the original one. The Gaussian smoothing option was selected, which smoothes all contours.

### Linear measurements for the segmented condyle

**Fig. 8:**
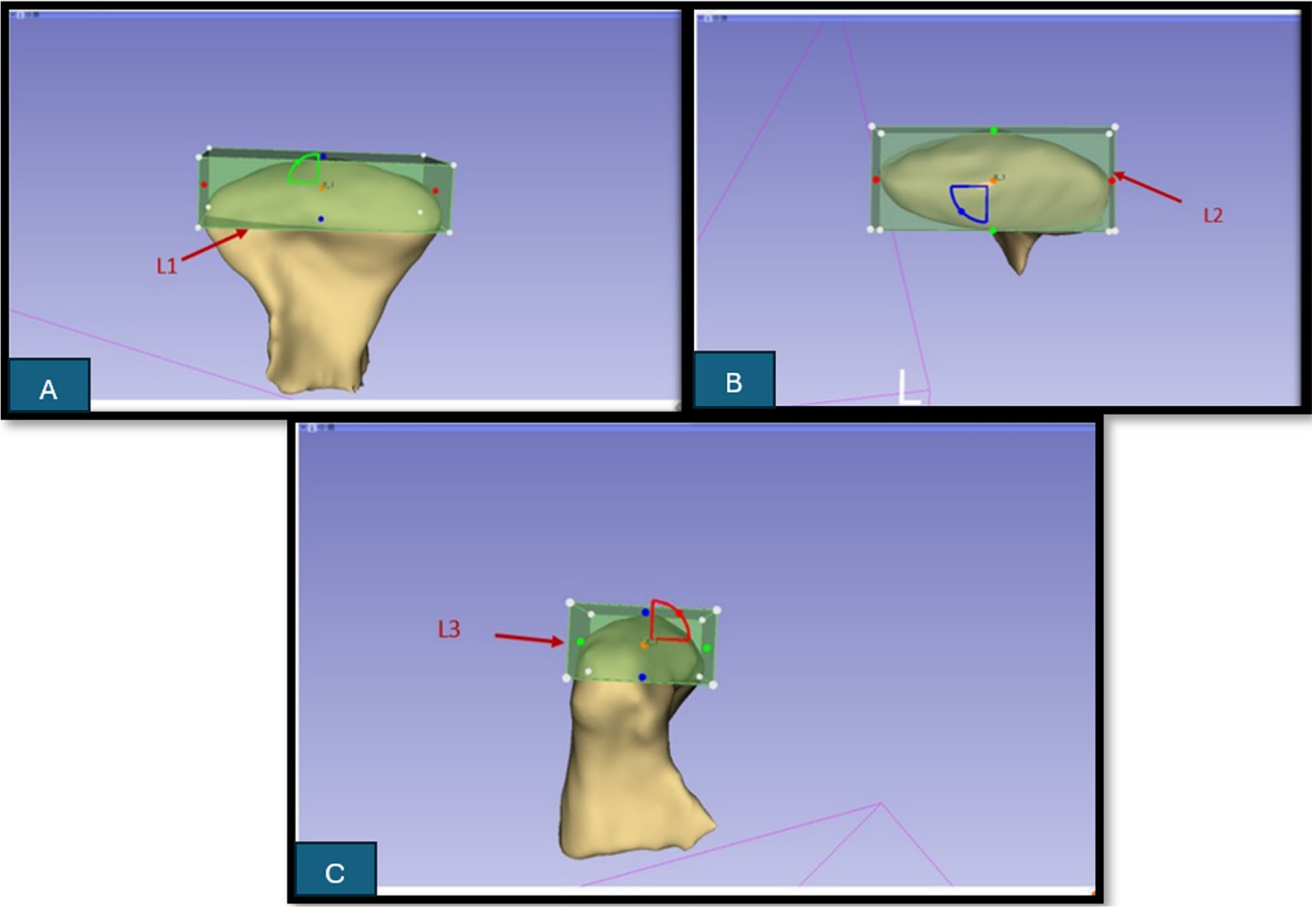
Screenshots of the software showing the linear measurements methods **A**: A screenshot from the software showing the 3D rectangle generated from the “ROI” option in the “Markups” module and showing the aspect of the horizontal longest sides of this box which represent L1 (Condylar width). **B**: A screenshot from the software showing the horizontal shortest sides of the box which represent L2 (Condylar length). **C**: A screenshot from the software showing the vertical sides which represent L3(Condylar height).The cuboidal ROI corresponded to the actual caliper measurements

On the 3D condylar volume generated either by manual or semiautomatic segmentation, the “Markups” module was activated, on which a 3D cuboidal region of interest, “ROI” was selected and adjusted over the condyle in a way that the horizontal longest sides of this cuboid represented L1 measurement (*Condylar width)* (X) (Fig. 8A). The horizontal shortest sides of the cuboid represented the L2 measurement (*Condylar length)* (Z) (Fig. 8B), and the vertical sides represented the L3 measurement (*Condylar height)* (Y) (Fig. 8C). L1, L2, and L3 calculated by the software were recorded and tabulated. The software represents the sides of the 3D cuboidal region of interest as follows: The L-R range represents the horizontal longest side of the box. The P-A range represents the horizontal shortest side of the box. The I-S range represents the vertical sides. The longest and shortest horizontal sides of the cuboidal ROI corresponded to the actual caliper measurements for L1 and L2; however, the vertical side representing condylar height did not accurately replicate the real engagement of the caliper tips as achieved for L1 and L2.

It is worth mentioning that the radiographic linear measurements derived from the 3D cuboidal region of interest (ROI) reflect geometry-based bounding dimensions of the segmented condyle rather than true anatomical landmark-to-landmark distances. The software-generated values represent the maximal extent of the condyle along the orthogonal X, Y, and Z axes of the ROI. Accordingly, these CBCT-derived measurements should be interpreted as geometric approximations of condylar dimensions.

All the radiographic assessment was done by two oral radiologists with different experiences ranging from 5 to 18 years. Each observer was blinded to the real physical measurements and the results of the other observer. The second observer evaluated 33% of the total sample size for inter-observer assessment.

One of the two oral radiologists had done the assessment twice, with a two-week interval between the two sessions to assess intra-observer agreement.

### Statistical analysis

Numerical data were presented as mean with 95% confidence intervals (CI), standard deviation (SD), minimum (min), and maximum (max) values. They were tested for normality using Shapiro-Wilk’s test. Data were normally distributed and were analyzed using repeated measures ANOVA followed by Bonferroni post hoc test. Reliability was analyzed using the intraclass correlation coefficient (ICC) based on single measurements and absolute agreement. Method agreement and intra-rater reliability were analyzed using two-way mixed-effects models, while inter-rater reliability was analyzed using a two-way random-effects model. The significance level was set at *p* < 0.05 within all tests. Statistical analysis was performed with R statistical analysis software version 4.3.2 for Windows.

## Results

Results are illustrated in Tables 1-4

**Table 1:** Intergroup comparisons of the real, semi-automatic and manual linear measurements (Condylar width (L1), condylar length (L2), condylar height (L3) and all linear measurements collectively) in mm

Measurement	Linear measurementa (Mean ± SD)			*p*-value
	Real	Software (semiautomatic)	Software (manual)	
L1 (mm)	19.93 ± 1.96B	19.33 ± 1.99 C	20.33 ± 1.75 A	< 0.001*
L2 (mm)	9.90 ± 1.22B	10.48 ± 1.23 A	10.64 ± 1.16 A	< 0.001*
L3 (mm)	6.43 ± 1.06 A	5.83 ± 0.56B	6.22 ± 0.63AB	0.007*
Linear measurements collectively(mm)	12.08 ± 0.99B	11.88 ± 0.91B	12.40 ± 0.85 A	< 0.001*

Values with different superscript letters within the same horizontal row are significantly different, * Significant (*p* < 0.05)

**Table 2:** Agreement analyses for software measurements in both manual and semi-automatic segmentation with real measurements considering L1, L2, L3 and linear measurements collectively

Measurement	ICC (95% CI)	
	Real-Software (semiautomatic)	Real-Software (manual)
L1 (mm)	0.901 (0.741:0.957)*	0.971 (0.850:0.990)*
L2 (mm)	0.777 (0.433:0.903)*	0.837 (0.075:0.949)*
L3 (mm)	0.067 (-0.639:0.511)	0.466 (-0.108:0.744)*
Linear measurements collectively (mm)	0.881 (0.747:0.943)*	0.921 (0.696:0.970)*

* Significant (p<0.05)

**Table 3:** Intergroup comparison between the measurement error of the semiautomatic and manual segmentation techniques for L1,L2,L3 and linear measurements collectively in mm

Measurement	Mean ± SD		*p*-value
	Software measurements (semiautomatic)	Software measurements (manual)	
L1 (mm)	0.80 ± 0.62	0.54 ± 0.32	0.163
L2 (mm)	0.90 ± 0.63	0.89 ± 0.40	0.917
L3 (mm)	1.02 ± 0.81	0.76 ± 0.71	0.080
Linear measurements collectively (mm)	0.46 ± 0.44	0.44 ± 0.27	0.871

* Significant (*p* < 0.05)

**Table 4:** Inter-observer reliability and Intra-observer reliability using the intraclass correlation coefficient

Measurement	ICC(95%CI)
Inter-observer reliability	0.997 (0.995:0.998)*
Intra-observer reliability	0.998 (0.996:0.999)*

* Significant (*p* < 0.05)

## Discussion

Over the last decade, assessment of the mandibular condyle has been of interest to many researchers as it has a complex structure that affects the growth and function of the jaws, plus being an integral part of the TMJ, the sole joint in the maxillofacial region. Assessment of the morphology, integrity, and structural changes of the osseous components of the TMJ has been accomplished by different modalities such as panorama, linear or complex motion tomography, CBCT, and multi-slice CT [11]. CBCT is anticipated to be the ideal technique in TMJ osseous components assessment because of radiation dose reduction and greater convenience [11].

CBCT offers 3D volumetric assessment of the condyle, which is a complex anatomical structure of great importance in maxillofacial dentistry. The mandibular condyle significantly affects the mandibular growth, therefore morphological and dimensional condylar changes can affect craniofacial growth. Furthermore, temporomandibular disorder (TMD), as well as various malocclusions, can be the consequences of improper mandibular condylar growth and development [2, 3]. Morphological and histological changes in the temporomandibular joint can be caused by age progression and functional disorders as well [4].

Skeletal relapse, due to alterations in the TMJ location and morphology, was commonly reported following orthognathic surgeries. Some studies found that these changes were attributed to physiological remodeling of the TMJ. On the contrary, postoperative occlusal instability and relapse, TMD, and progressive condylar resorption (PCR) were reported by other researchers. Consequently, evaluation of the condylar condition radiographically is essential to estimate the severity of the condition and to develop an appropriate treatment plan [12, 13].

Using CBCT data, a variety of methods have been implemented to render the condylar surface in three dimensions [14]. Manual and semiautomatic techniques are frequently used for the segmentation of the mandibular condyles [2, 8, 9, 14–16] .Manual segmentation has been considered the gold standard segmentation technique for the condyles as it facilitates the delineation of condyles despite their low density and their proximity to other structures [2, 11]. By having the operator define the structure in each slice, the manual segmentation technique achieves its high accuracy, although it is an extremely tedious and time-consuming procedure. Nevertheless, the semiautomatic segmentation technique depends on threshold-based or region-growing segmentation [1]. And this is faster than the manual technique, which is clinically significant [2].

3D slicer software is an open source segmentation software (http://www.slicer.org) which is a user friendly with comparable accuracy to other software programs (ITK-Snap, Invesalius Dolphin 3D), excellent intra-operator and inter-operator reliability in condylar segmentation as mentioned by Giudice et al [2]. Giudice et al [2] found that the volumetric measurements of the condylar models from 5 software programs (3D Slicer, ITK-Snap, Invesalius Dolphin 3D, and Mimics) showed no significant difference.

*Regarding L1 measurements (Condylar width)*, all post hoc pairwise comparisons were statistically significant (*p* < 0.001). However, the mean measurement error in semiautomatic technique measurements was (0.80 ± 0.62) (mm), which was higher than that measured in manual technique measurements (0.54 ± 0.32) (mm), yet the difference was not statistically significant (*p* = 0.163). Considering the clinically accepted error in most of the dental fields (0.5 mm) [2]. Both exceeded it, although the difference was minimal in the manual technique. On the other hand, the agreement analysis between both segmentation techniques, measurement, and the real gold standard measurements showed excellent agreement that was statistically significant in both (ICC > 0.9, *p* < 0.001).

*Regarding L2 measurements (Condylar length)*, Post hoc pairwise comparisons showed that real measurements had significantly lower values than other measurements (*p* < 0.001). The mean measurement error in semiautomatic technique measurements (0.90 ± 0.63) (mm) was almost comparable to that measured in manual technique (0.89 ± 0.40) (mm), and there was no statistically significant difference between them (*p* = 0.917). However, the error values in both exceeded the clinically accepted error in most of the dental fields. In the same way, the agreement analysis between both segmentation techniques, measurement, and the real gold standard measurements showed good agreement that was statistically significant in both (0.9 > ICC > 0.7, *p* < 0.001).

*Regarding L3 measurements (Condylar height)*, Post hoc pairwise comparisons showed that real measurements had significantly higher values than semiautomatic technique measurements (*p* < 0.001). The mean measurement error in semiautomatic technique measurements (1.02 ± 0.81) (mm) was higher than that measured in manual technique measurements (0.76 ± 0.71) (mm), yet the difference was not statistically significant (*p* = 0.080). And both errors exceed clinically accepted limits in most dental fields. In addition, the agreement analysis between the software and the real gold standard measurements showed poor agreement. Whereas the ICC for the semiautomatic and manual techniques was 0.067 and 0.466, respectively. The agreement was not statistically significant (*p* > 0.05) for the semiautomatic technique, which coincides with the lack of reproducibility. This could be explained based on surface irregularities in the upper surface of the condyle, which made the assessment of the condylar height not reliable adequately.

*Regarding the linear measurements collectively*, post hoc pairwise comparisons showed that manual technique measurements had significantly higher values than other measurements (*p* < 0.001). The mean measurement error in semiautomatic technique measurements (0.46 ± 0.44) (mm) was higher than that measured in manual technique measurements (0.44 ± 0.27) (mm), yet the difference was not statistically significant (*p* = 0.871), which is still below the clinically accepted error. For semiautomatic technique measurements, the agreement was good (ICC = 0.881, *p* < 0.001), while for manual technique measurements, the agreement was excellent (ICC = 0.921, *p* < 0.001).

Comparing our results with those reported by previous similar studies we can see that there was partial agreement with the error reported by García-Sanz et al. [10], who evaluated the reliability and accuracy of linear and volumetric measurements of mandibular condyles using the superimposition of surface models on CBCT images to isolate the mandibular condyle, where their mean difference between CBCT and gold standard was 0.9 ± 0.84 mm in condylar length (L2) which was very close to ours in the same type of condylar measurement. While a contradiction between us appeared in the condylar width (L1) and height(L3), where they reported a mean error of 0.04 ± 0.27 and 0.02 ± 0.54 mm, respectively, which is far below our reported error. This big difference could be attributed to the fact that García-Sanz et al. [10] measured the linear measurements on CBCT cross-sectional images unlike ours that were done on the 3D rendered images, and this explains their higher accuracy in the condylar width and height and their relatively lower accuracy in length measurements (that was comparable to ours) which is better achieved by mimicking the real 3D position of the condyle. Taking into consideration that they utilized a smaller voxel size (0.2 mm), which could invite a higher accuracy.

Accordingly, the similar accuracy observed for L1 and L2 measurements using manual and semi-automatic segmentation suggests that the segmentation method itself was not the main source of measurement differences. In contrast, the limitations seen for L3 appear to be largely related to the geometric constraints of the ROI-based measurement approach.

Kim et al.[1] used 3D printed mandible models, which were generated from a dry mandible, as a gold standard to validate a technique for mandibular condyle segmentation and volume determination by using CBCT. In line with our findings, the agreement between the semiautomatic technique measurements and those obtained from the physical replica was excellent (ICC = 0.998, *P* < 0.001). Their reported mean error was 0.51 ± 0.94 mm, which was close to our mean error reported in the linear measurements collectively.

Interpreting our results showed a higher variability for the semiautomatic technique, which might be explained on the basis that the semiautomatic segmentation process relies on spatial and contrast resolution of the scan, the thickness and degree of calcification or cortication of the bony structure, and the software algorithm. Considering the present findings, it is possible that software based on growing region algorithm as 3D Slicer may induce difficulties during the segmentation process. Since the seed points may not cover the hypodense voxels [2].

Important factors related to the imaging modality used (CBCT) should be considered while interpreting our results, which are the intrinsic low contrast resolution, partial volume effect, lower signal-to-noise ratio, and distortion of Hounsfield Units (HU-value). These drawbacks hampered the accuracy of the segmentation process, especially with the semiautomatic technique, which depends significantly on the grey scale values obtained from CBCT (distorted HU-value) [8]. In addition, the spatial resolution of the condylar areas is more affected due to higher noise in the peripheral areas of the CBCT scan [2].

Our study was designed to evaluate and analyze condylar linear measurements in a reliable and reproducible manner in order to bridge the gap of knowledge highlighted by Kim et al. (2020) [17]. In his systematic review, which concluded that the semiautomatic segmentation from CBCT images is a reliable condylar segmentation method, but more studies are required to assess its accuracy against a validated reference standard. For that, in our study, we tried to standardize the exposure factors, scanning protocol, the limits of both linear condylar measurements in both real and CBCT measurements, and the software used. Furthermore, to ascertain the reliability of our results, one radiologist evaluated the radiographs 2 times with a time lag of two weeks to guarantee intra-observer reliability, and a 2nd observer evaluated 33% of the cases for inter-observer reliability agreement. For all measurements, there was an excellent agreement in both intra-observer and interobserver reliability (ICC > 0.9).

Despite the meticulous methodological settings previously discussed, there were some unavoidable limitations we faced in this study, which were:

- 1- L3 measurement was not reproducible due to the condylar surface irregularities.
- 2- The limitation of the imaging modality used.
- 3- Separation between the condylar surface and the glenoid fossa was sometimes not fully achieved by the piece of cloth used which made the segmentation difficult.
- 4- The cuboidal ROI-based linear measurements represent geometric approximations of condylar dimensions rather than direct anatomical landmark-to-landmark distances.

From the results of this study, we concluded that semiautomatic segmentation of the mandibular condyle is a reliable and time-saving segmentation approach that yields accurate results comparable to those of manual segmentation in both condylar length and width measurements, whereas for the condylar height measurements, higher discrepancy in accuracy was noted with both segmentation techniques. These findings indicate that measurement accuracy in CBCT-derived condylar assessment is influenced not only by the segmentation technique but also, and in some dimensions predominantly, by the measurement framework used to extract linear dimensions from three-dimensional segmented volumes.”

And it is recommended that more studies should be conducted to assess the semiautomatic technique against a real physical reference. And because of the new era of artificial intelligence, more studies are required to assess its accuracy in mandibular condyle segmentation

## Data Availability

The datasets used and/or analyzed during the current study are available from the corresponding author on reasonable request.

## Abbreviations

3D: Three dimensional
ICC: Intraclass correlation coefficient
TMJ: Temporomandibular joint
MRI: Magnetic resonance imaging
CT: Computed tomography
CBCT: Cone beam computed tomography
TMD: Temporomandibular disorder
ROI: Region of interest
PCR: Progressive condylar resorption
CI: Confidence interval
SD: Standard deviation
min: minimum
max: maximum
HU: Hounsfield unit
